# Concurrent Cortical Representations of Function- and Size-Related Object Affordances: An fMRI Study

**DOI:** 10.3758/s13415-018-0633-1

**Published:** 2018-08-28

**Authors:** Dimitrios Kourtis, Pieter Vandemaele, Guy Vingerhoets

**Affiliations:** 1 Psychology, Faculty of Natural Sciences, University of Stirling, Stirling, Scotland FK9 4LA UK; 2 Department of Experimental Psychology, Ghent University, Ghent, Belgium; 3 Department of Radiology, Ghent University, Ghent, Belgium

**Keywords:** Object affordances, Object size, Object function, Dorsal stream, Posterior parietal cortex, fMRI

## Abstract

Previous work has shown that the perception of a graspable object may automatically potentiate actions that are tailored to specific action-related features of the object (e.g., its size) and may be related to its immediate grasping as well as to its long-term, functional use. We investigated the neural correlates of function- and size-related object affordances that may be concurrently potentiated by a graspable object. Participants were lying in a MR scanner holding a large switch in one hand and a small switch in the other hand. They passively attended a large or a small object with clearly separated functional and graspable end that was displayed centrally at an average angle of 45 degrees. Participants responded to the direction of an arrow that was overlaid on the object after a mean period of 1,000 ms after object onset and was pointing to the left or to the right with equal probability. Response times were shorter when the arrow pointed to the functional end of the object and when the responses were made with the switch that was congruent to the size of the perceived object. A clear distinction was found in the representation of function- and size-related affordances; the former was represented in the posterior parietal cortex and the latter in prefrontal, premotor, and primary sensorimotor cortices. We conclude that different aspects of object-directed actions may be automatically potentiated by individual object features and are represented in distinct brain areas.

## Introduction

The particular status of tools for human behavior is not only reflected by how we reason about them but also by how our brain responds to tool stimuli (Vaesen, 2012). Compared with other object categories, such as animals, houses, or faces, tool stimuli elicit extra-temporal activation in frontal and parietal regions that have been associated with motor processes (Chao et al., 1999; Chao and Martin, 2000; Creem-Regerh and Lee, 2005). Remarkably, tools seem to activate these motor-related regions automatically, that is, upon passive observation of a viewer who has no intention to interact with the perceived tool. This automaticity is akin to the phenomenon of motor affordance, in which the mere perception of a graspable object facilitates motor responses that are congruent with certain qualities of the object, such as its size or orientation (Ellis and Tucker, 2000; Phillips and Ward, 2002; Tucker and Ellis, 1998, 2001). At a clinical level, the automatic nature of uninhibited motor affordances can be observed in patients with manual groping or utilization behavior that compels the patient’s hand to follow, grasp, or use objects (Lhermitte, 1983).

The potentiation of an action that is afforded by an object may occur automatically (Tucker and Ellis, 1998), but it also depends on a number of factors, such as the allocation of attention to the object as a whole (Riggio et al., 2008) or to an action-relevant feature of the object (Pellicano et al., 2010), the hand shaping of the prospective actor (Ansuini et al., 2008), and also the graspability (Symes et al., 2007) and the reachability of the object (Costantini et al., 2010). Multiple features of an object can be processed in parallel (Duncan, 1984) and can induce concurrent representation of actions, which may be related to different structural features and/to the skillful use of the object (i.e., “structural/volumetric affordances vs. “functional affordances”) (Binkofski and Buxbaum, 2013; Bub et al., 2008; Jax and Buxbaum, 2010). Object affordances also can be categorized on the basis of whether they are derived from time-invariant or from temporary features of an object (see “stable” vs. “variable” affordances) (Borghi and Riggio, 2009, 2015).

The objective of our experiment was to investigate, by means of functional magnetic resonance imaging (fMRI), the neural correlates of action representations that are related to the immediate grasping (i.e., size-related affordances) and the long-term skillful use (i.e., function-related affordances) of an object—when a person pays attention to the object but has no intention to act upon it. Our experimental design was based on the designs used in the Tucker and Ellis (1998) and Ellis and Tucker (2000) studies, which showed that object-directed actions are intrinsic to the representation of an object. An object, either a large or a small one, was presented centrally on a computer monitor, tilted at an average angle of 45° (range: 30-60°) and in such a way that the graspable part of the object was located at the lower (left or right) quadrant of the monitor and the functional part of the object at the opposite upper quadrant. It should be noted that we use the term “functional part” to refer to the part of the object that signifies the object’s function/identity (e.g., the blade of a knife, the head of a hammer, etc.). Participants were lying in the MR scanner holding a large response switch in one hand and a small response switch in the other hand. The participants were not instructed to attend specific features of the object but rather to attend the object as a whole and only respond to the direction of an arrow that was overlaid on the object after an average time interval of 1,000 ms after object onset. The response was made by pressing the switch that was spatially congruent to the direction of the arrow. We hypothesized that the size of the displayed object would automatically activate the representation of the corresponding grasping action. Such representation should facilitate a size congruent response and interfere with a size incongruent response. For example, the display of a small object should activate the representation of a precision grip, and the participants should be faster at responding with the small switch (held with a precision grip) compared to responding with the large switch (held with a power grip). With regards to the potentiation of action related to the long-term functional use of an object, we drew our predictions from a recent EEG study with a similar experimental design and stimuli (Kourtis and Vingerhoets, 2015), which showed that the functional part of the object (e.g., the head of a hammer) first captured a passive observer’s attention and then potentiated a spatially compatible response (see also, Bub et al., 2008; Pellicano et al., 2010). Accordingly, we expected that the participants would be faster responding to an arrow pointing to the functional end of a displayed object.

Previous neuroimaging studies have demonstrated that the posterior parietal cortex (PPC) is integral in object perception, planning of object-directed actions, and in the representation of visuomotor affordances (Grèzes and Decety, 2002; Grèzes et al., 2003; Vingerhoets, 2008, 2014). The PPC is part of the dorsal processing stream of visual information, which is traditionally associated with spatial representation/localization (“where”) and movement guidance (“how”), and it is functionally complementary to the ventral processing stream, which is associated with abstract knowledge about objects and their spatial relationships (“what”) (Goodale and Milner, 1992; Milner and Goodale, 2008; Ungerleider and Mishkin, 1982). More recently, Rizzolatti and Matelli (2003) put forward the theory that the dorsal stream may consist of two distinct pathways: a dorso-dorsal pathway, which is responsible for the online control of action (the “Grasp” system, related to structure-based affordances), and of a ventro-dorsal pathway responsible for the purposeful use of objects based on stored knowledge (the “Use” system, related to function-based affordances) (Binkofski and Buxbaum, 2013; Buxbaum and Kalenine, 2010). Such distinction is also consistent with the conceptual categorization of affordances on the basis of the temporal (in)variability of object features (Borghi and Riggio, 2009). “Stable” affordances that are driven by existing knowledge of an object’s time-invariant features seem to be predominantly represented in the ventro-dorsal pathway, whereas “variable” affordances that are related to the processing of temporary object features (e.g., its orientation) seem to be represented in the dorso-dorsal pathway (Sakreida et al., 2016).

In addition to PPC, passive viewing of a graspable object activates occipital and temporal areas—associated with object recognition and identification processes (Milner and Goodale, 1995), prefrontal areas, associated with decision-making processes, and more importantly premotor and primary motor areas (Chao and Martin, 2000; Grèzes and Decety, 2002; Grèzes et al., 2003; Valyear et al., 2012; Vingerhoets et al., 2013). Such activation patterns indicates that passive viewing of an object does not only involve the processing of its visual properties and its identity, but it also induces the representation of actions that are afforded by specific features of the object. Accordingly, we expected that the display of a graspable object would activate an extended cortical network associated with immediate object grasping and long-term skillful use and we sought to examine whether these two type of action affordances are represented in the same or distinct brain areas.

## Methods

### Participants

Twenty-two healthy volunteers participated in the study. Two participants were excluded from the analyses due to head movements that exceeded the size of a functional voxel (>3 mm), and an additional participant was excluded due to unstable handling of the large response switch. The 19 remaining participants were 11 women and 8 men with a mean age of 22.2 (range: 19-27) years. All were right-handed as determined by the Edinburgh Handedness Inventory: *M* = 89.7%, *SD* = 12.6% (Oldfield, 1971), and none had a history of neurological or psychiatric disease. Scanning protocols were approved by the local ethics committee, and all subjects gave written, informed consent after the experimental procedure had been explained.

### Procedure

**Fig. 1:**
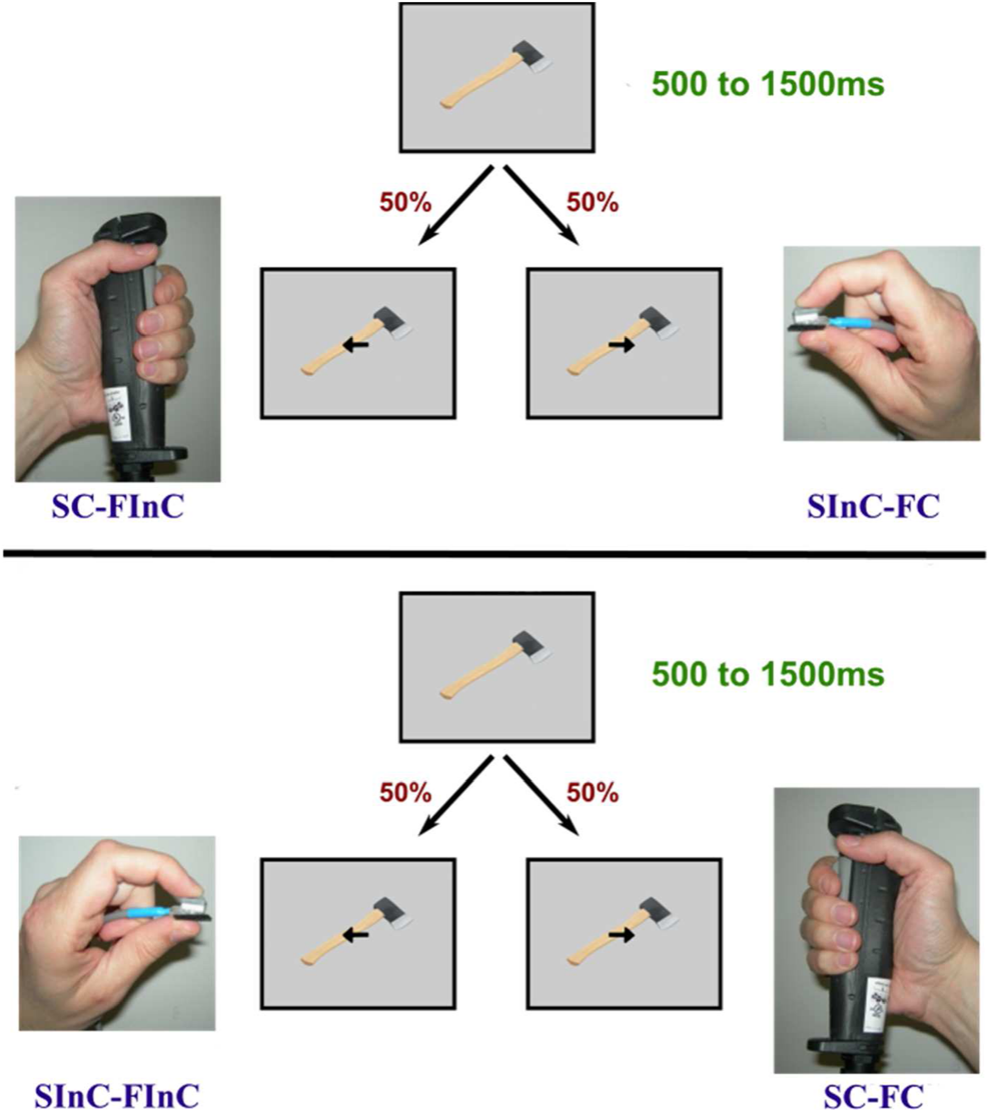
Two examples of experimental trials. Top: The participant holds the small switch with the right hand and the large switch with the left hand. A large object is displayed on the screen for a variable time period, ranging from 500 to 1500 ms, after which an arrow is overlaid on the object pointing either left or right. Responses with the left hand would be size congruent (large object – large switch) but function incongruent, because the arrow points away from the object’s handle. Conversely, responses with the right hand would be size incongruent but function congruent. Down: The participant holds the large switch with the right hand and the small switch with the left hand. In this case, responses with the left hand would be size incongruent and function incongruent, whereas a responses with the right hand would be size congruent and function congruent

Before scanning, the participants completed a pre-scan MRI-safety questionnaire and the Edinburgh Handedness Inventory and provided informed consent for their participation in the study. Next, the participants were positioned head first and supine in the magnet with their left and right arms placed alongside the body on the scanner table. The participants were holding a large MR-compatible response switch using a power grip in one hand (Fig. 1, left) and a small MR-compatible response switch using a precision grip in the other hand (Fig. 1, right). Of the 19 remaining participants, 11 held the large switch with their right hand and the small switch with their left hand, and 8 held the large switch with their left hand and the small switch with their right hand.

The participants’ heads were gently fixed in place with foam cushions. Participants were reminded of the fact that MR imaging is very sensitive to movement and that they were required to restrict head movements and to lie as still as possible. Stimulus presentation was controlled using Presentation software (Neurobehavioral Systems Inc., Berkeley, CA) synchronized with the MRI scanner. The stimuli were back projected on a screen at the back of the magnet bore and viewed via a mirror attached to the head coil. After the end of the experiment, the participants completed a post-scan MRI safety questionnaire and were debriefed regarding the purpose of the study.

### Experimental Paradigm

Each experimental trial started with the display of a photo of a small (e.g., key, pin, etc.) or a large object (e.g., axe, screwdriver, etc.). The object was displayed centrally and titled at an angle of an average of 45° (range: 30-60°), with its graspable part located either at the left or at the right lower visual quadrant with equal probability and its functional part at the opposite higher quadrant. The size of the objects varied between 12 and 20 cm for the large objects and between 4 and 8 cm for the small objects for them to look graspable either with a power grip (large objects) or with a precision grip (small objects), within reach to the participants and also to correspond to a large extent to their actual average size in everyday life (e.g., a spoon cannot be the same size as a hammer). The duration of an object’s display varied randomly between 500 and 1,500 ms with a step increment of 250 ms, after which a black arrow was overlaid on the object. The arrow pointed to the left or to the right with equal probability. The participants were instructed to gaze at the centre of the screen and pay attention to the object. No specific instruction was given related to the part or feature of the object that they had to attend to. They also were asked to respond swiftly by pressing the switch, which was held at the side pointed by the arrow head. After the participants’ response, the stimulus was replaced by a centrally presented fixation cross, which remained on display until the onset of the following cue stimulus. The time interval between the arrow onset and the start of the next trial was 2,000 ms.

There were 50 large and 50 small objects in total (Table 1). Each object was presented four times: twice with the functional part on the left side and twice on the right side. To ensure that the participants were paying attention to the object, we introduced “catch” trials, consisting of the photo of an animal, to which participants were instructed to respond immediately with both hands. There were 20 animals in total; each was presented twice, with its head either on the left or right side. Overall, the experiment consisted of 440 randomly presented trials (i.e., 100 trials/condition), 40 of which were catch trials.

**Table 1:** List of objects that were used as stimuli

Small objects		Large objects	
Binder clip 1	Binder clip 2	Axe	Bottle opener 1
Binder clip 3	Small glue tube	Bottle opener 2	Bottle opener 3
Hairclip 1	Hairclip 2	Long brush 1	Long brush 2
Bobby pin 1	Bobby pin 2	Long brush 3	Hair brush
Key 1	Key 2	Clothes brush	Cable stripper 1
Key 3	Key 4	Cable stripper 2	Meat clever
Lipstick 1	Lipstick 2	Egg beater	Frying pan
Nail clipper 1	Nail clipper 2	Garlic crusher	Hammer 1
Nail clipper 3	Nail clipper 4	Hammer 2	Hammer 3
Eyebrow tweezers 1	Eyebrow tweezers 2	Hammer 4	Drill
Pen 1	Pen 2	Ice cream scoop	Spatula 1
Match 1	Match 2	Spatula 2	Spatula 3
Nail 1	Nail 2	Nut cracker	Paper cutter
Nail 3	Clothes peg 1	Ripping pincher	Pliers 1
Clothes Peg 2	Memory stick	Pliers 2	Pliers 3
Push pin 1	Push pin 2	Saw 1	Saw 2
Push pin 3	Eyebrow tweezers	Saw 3	Screwdriver 1
Needle 1	Needle 2	Screwdriver 2	Screwdriver 3
Needle 3	Screw 1	Letter opener	Sickle
Screw 2	Screw 3	Pasta Ladle	Wrench 1
Screw 4	Coffee stirrer 1	Wrench 2	Wrench 3
Coffee stirrer 2	Coffee stirrer 3	Wrench 4	Trowel
Pencil 1	Pencil 2	Knife 1	Knife 2
Paperclip 1	Paperclip 2	Knife 2	Potato masher
Small Allen key	Slim tin opener	Chisel 1	Chisel 2

**Fig. 2:**
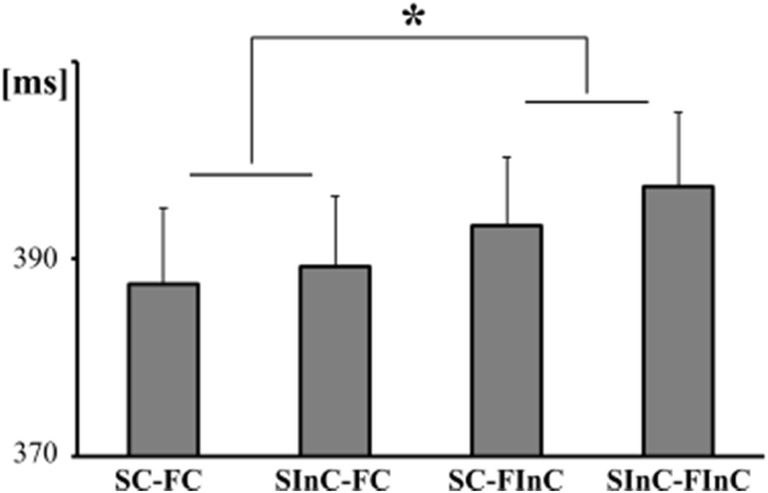
Response times. SC, size congruent; SInC, size incongruent; FC, function congruent; FInC, function incongruent. The asterisk depicts the significant function-related affordance effect. The error bars represent standard error of mean

We investigated two types of response congruency based on two different features of an object (Fig. 2). The first type was size congruency, which refers to the correspondence between the size of the object and the size of the response switch. For example, when a large object was on display, a size congruent (SC) response would be the pressing of the large switch and a size incongruent (SInC) response the pressing of the small switch. The second type was spatial congruency, which refers to the correspondence between the location of the functional part of an object (e.g., the blade of a knife) and the location of the responding hand. To distinguish it from the spatial congruency that refers to the correspondence between the locations of the graspable part of an object (e.g., the handle of a knife) and the location of the responding hand, we will use the term “function congruency.” For example, when the functional part of the object was located at the right side, a function congruent (FC) response would be a right-hand response and a function incongruent (FInC) response would be a left-hand response. Based on these different types of congruency, we defined four experimental conditions in which a response could either be: i) size congruent and function congruent (SC-FC); ii) size congruent and function incongruent (SC-FInC); iii) size incongruent and function incongruent (SInC-FC); or iv) size incongruent and function incongruent (SInC-FInC).

### Scanning procedure

Scanning was performed at 3.0 T on a Siemens Trio MRI scanner (Siemens Medical Systems, Erlangen, Germany) that was equipped with echo planar imaging (EPI) capabilities and used a 32-channel head coil for radio frequency transmission and signal reception. After automatic shimming of the magnetic field on each participant, a 3-D high-resolution T1 anatomical image of the whole brain in the sagittal plane was acquired for co-registration with the functional images (3D MPRAGE, 176 slices, slice thickness = 1.0, in-plane resolution = 1.0 x 1.0 mm, TR = 2250 ms, TE = 4.18). Next, 590 functional EPI images in the axial plane were acquired. They had the following parameters: TR = 2.5 s, TE = 27 ms; flip angle = 62°, 33 slices, slice thickness = 2.5 mm, slice gap = 1.25 mm, FOV = 192 mm and matrix = 64 x 64, resulting in a resolution of 3 x 3 x 2.5 mm.

### Behavioral Analysis

The primary focus of our analyses was the Response Time (RT), which was defined as the time interval from the onset of an arrow stimulus until the press of a response switch. Trials where the participants did not respond or pressed the wrong switch or where they were too fast or too slow to release the response keys (i.e., the difference with the mean RT was larger than 2 standard deviations in either direction) were removed from subsequent RT analysis. In addition, we examined the participants’ errors in cases where the participants failed to respond or pressed the incorrect switch.

### Image analysis

Data analysis was performed using Brain Voyager QX for pre-processing and statistical inference (Goebel et al., 2006). Functional data were subjected to a standard sequence of pre-processing steps comprising slice scan time correction, 3-D motion correction by spatial alignment to the first volume, and temporal filtering using linear trend removal and high pass filtering for low frequency drifts of three or fewer cycles. Spatial smoothing with a Gaussian filter (FWHM = 8 mm) was applied for the volume-based analysis. The anatomical data for each subject were transformed into Talairach standard space using sinc interpolation. The functional data for each subject were co-registered with the subject’s 3-D anatomical dataset and transformed into Talairach space.

The volume time course was then subjected to an independent component analysis (ICA) to define noise predictors for use in the General Linear Model (GLM) design matrix. For each participant, a protocol file representing the onset and duration of each event for the different conditions was derived. Factorial design matrices were defined automatically from the created protocols. The BOLD response in each condition was modeled by convolving the defined conditions with a canonical hemodynamic response function (gamma) to form the main predictors in the GLM. After exclusion of potentially task-related components (r >|0.30|), we added the remaining ICA components as predictors-of-no-interest to the GLM design matrix. Finally, we applied a cortex-based mask that was created from the averaged anatomical scans of all volunteers. After the GLM had been fitted, t-maps were generated to evaluate the effects of relevant contrasts. As we expected between-contrast differences to be subtle, we used cluster-based inference to correct for multiple comparisons. The method exploits the fundamental assumption that areas of activity tend to stimulate signal changes over spatially contiguous groups of voxels rather than over sparsely isolated voxels. A minimum cluster size of 10 voxels (at *p* = 0.005 (uncorrected)) has been recommended by some authors (Lieberman and Cunningham, 2009), but we applied a more stringent combination of an uncorrected threshold of *p* < 0.001 in all analyses with a minimal voxel threshold of 27 voxels (3 x 3 x 3 mm). This threshold setting resulted in a minimal t-value of 3.92 for a voxel to be considered significant. In short, we consider the likelihood that 27 adjacent voxels being active at or above this threshold is above chance in a whole brain analysis investigating differences between slightly different conditions.

We sought for clusters of significant activation for comparisons between function congruent (FC) trials versus function incongruent (FInC) trials and for size congruent (SC) trials versus size incongruent (SInC) trials. Thus, we examined four different contrasts: i) SC > SInC; ii) SInc > SC; iii) FC > FInC; and iv) FInC > FC.

## Results

### Response Times

The response times (RTs) were 387.4 ms (SD = 30.3) for SC-FC responses, 389.2 ms (SD = 33.0) for SInC-FC responses, 393.4 ms (SD = 33.5) for SC-FInC responses, and 397.3 ms (SD = 31.2) for SInC-FInC responses (Fig. 2). A preliminary analysis showed that there was no difference between holding the large switch with the right hand and the small switch with the left hand compared to the opposite configuration (*p* = 0.908). Statistical comparisons were made using a 2 x 2 x 2 repeated measures ANOVA, with the factors: Switch size (large vs. small switch), Size Congruency (SC vs. SInC), and Function Congruency (FC vs. FInC). The analysis revealed a significant main effect of Switch Size [*F*(1,18) = 36.5, *p* < 0.001, *η*_*p*_^*2*^ = 0.670], because responses with the small switch were significantly faster. More importantly and related to the objectives of our study, there was a significant main effect of Function Congruency [*F*(1,18) = 12.5, *p* = 0.002, *η*_*p*_^*2*^ = 0.410], because participants were faster when they responded with the hand at the side of the functional part of the object. Moreover, the participants were faster to respond with the switch that corresponded with the size of the displayed object; however, the main effect of Size Congruency only showed a trend towards statistical significance [*F*(1,18) = 3.3, *p* = 0.087, *η*_*p*_^*2*^ = 0.154]. There was however a significant two-way interaction between Switch Size and Size Congruency [*F*(1,18) = 9.2, *p* = 0.007, *η*_*p*_^*2*^ = 0.338]. We investigated the origin of this interaction with post-hoc *t*-tests (Bonferroni corrected alpha level = 0.025), which showed that there was a significant effect of Size Congruency when participants were responding with the small switch [*t*(18) = −3.6, *p* = 0.002], but no effect when responding with the large switch [*t*(18) = 1.2, *p* = 0.231].^1^ No other interaction was statistically significant (*p*s > 0.12). The number of erroneous responses was extremely small (1.8%); thus, no statistical analysis was performed on the number of errors.

Neuroimaging Results

**Fig. 3:**
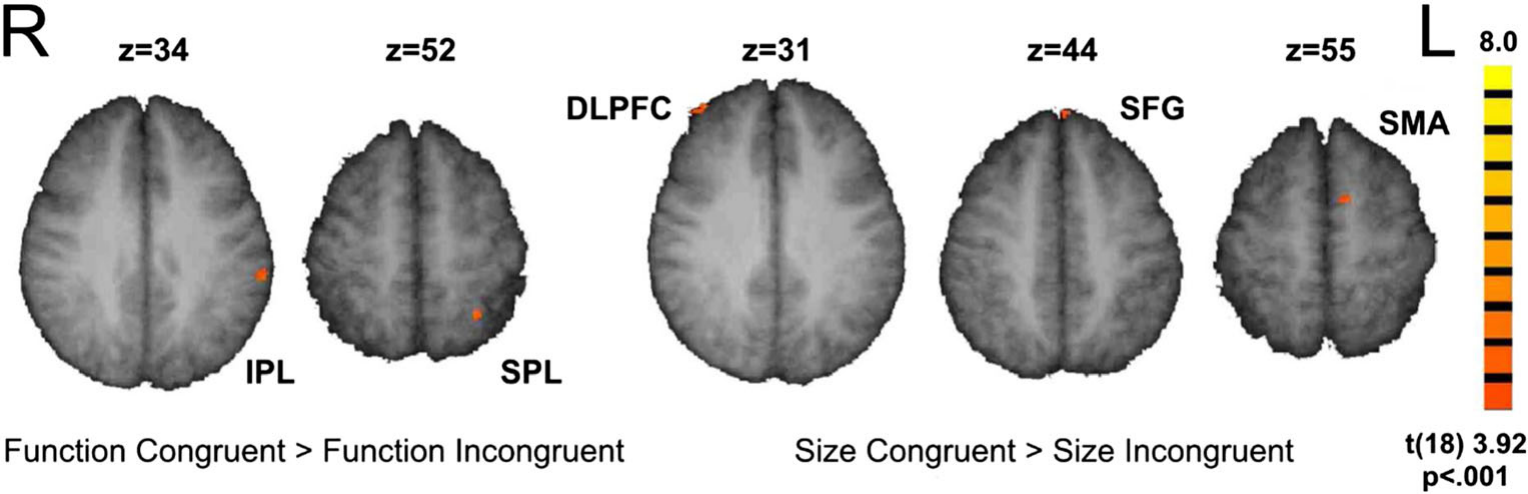
Most important fMRI contrasts. Function-related affordances and size-related affordances are represented in distinct brain areas, the former in posterior parietal cortex and the latter in prefrontal and premotor cortices

All four possible main contrasts related to size and function congruency (i.e., SC > SInC, SInc > SC, FC > FInC and FInC > FC) resulted in activation of occipital areas (Brodmann areas (BA) 18 and 19). In addition, the (FC > FInC) contrast revealed two clusters of activation in the left posterior parietal cortex (PPC): one cluster in the superior parietal lobule (SPL, BA 7) and another cluster in the inferior parietal lobule (BA 40). The opposite contrast revealed activation in the left posterior cingulate gyrus (BA23) and the left cerebellum (Lobes IV and V). Interestingly, PPC activation was not present in the (SC > SInC) contrast, but instead we observed activation in the right superior frontal gyrus (BA 9), in the medial superior frontal gyrus (BA 8), in the left medial frontal gyrus (BA 6), and also in the boundary between left precentral and postcentral gyri (BAs 4 and 3) (see Figure 3 for FC > FInC and SC > SInc contrasts). The opposite contrast (SInC > SC) revealed activation in the left dorsal premotor cortex (BA 6) (Figure 3; Table 2).

**Table 2.** Peak voxel coordinates in Talairach space of significant activation clusters of all statistically significant contrasts

	Brain Area	BA	x	y	Z	t_max_	# of voxels
SC > SInC	Left cuneus	**18**	-6	-97	13	8.19	2434
	Right lingual gyrus	**18**	15	-73	-8	6.96	1919
	Right cuneus	**18**	27	-98	4	5.47	107
	Left pre/postcentral gyrus	**4,3**	-36	-25	61	5.07	63
	Right superior frontal gyrus	**9**	36	47	31	5.06	28
	Left lingual gyrus	**17**	-12	-91	-11	4.97	51
	Left medial frontal gyrus	**6**	-9	-11	55	4.75	40
	Superior frontal gyrus	**8**	0	50	44	4.64	46
SInC > SC	Right middle occipital gyrus	**18**	12	-94	19	11.07	2799
	Left fusiform gyrus	**18/37**	-39	-76	-14	8.06	144
	Left lingual gyrus	**18**	-6	-76	-5	6.13	782
	Let middle frontal gyrus	**6**	-27	11	55	4.80	35
	Left fusiform gyrus	**19**	-30	-67	-8	4.51	39
FC > FInC	Left superior parietal lobule	**7**	-27	-52	52	5.42	40
	Left inferior parietal lobule	**40**	-60	-34	34	4.57	135
	Left lingual gyrus	**18**	-15	-82	-11	4.48	99
	Right middle occipital gyrus	**19**	27	-85	16	4.84	132
FInC > FC	Left posterior cingulate gyrus	**23**	-6	-58	10	5.08	32
	Left cuneus	**19**	-15	-97	23	4.74	99
	Cerebellum (IV,V)		-3	-40	-8	5.15	35

SC, size congruent; SInC, size incongruent; FC, function congruent; FInC, function incongruent

## Discussion

### Behavioral Results: the “function congruency” and “size congruency” effects

Our behavioral analysis revealed the presence of two distinct affordance effects, which emerged without interacting with each other: the “function congruency” effect and the “size congruency” effect. The first was related to location of the feature of the object that indicated the function/identity of the displayed object. The participants were faster to respond to an arrow pointing to the functional end of the object, which was located at the opposite side of the object’s grasping part (i.e., the handle). Although this finding may seem counterintuitive, it is a replication of a recent EEG study (Kourtis and Vingerhoets, 2015) with a very similar design to the present study, which differed mainly in that there was no size manipulation (the participants in that study responded by pressing keys on a computer keyboard). This strongly suggests that the “function congruency” effect should not be attributed to the complexity of the task (i.e., to the concurrent processing of multiple object features) but rather that it is a genuine effect showing that the processing of an object’s functional part receives priority over the processing of its graspable part.

It also may be argued that this effect simply reflects the directing of attention towards the most perceptually salient feature of an object and consequently the activation of a response code that is spatially compatible with the location of that feature (Anderson et al., 2002). Although this sounds plausible (e.g., the functional end of an axe is indeed its more salient part), it becomes less convincing when we consider the small objects that were used in our study, the functional part of which was most often their less salient part (e.g., keys, pins, nails, etc.). A more likely interpretation is that the “function congruency” effect does not depend on saliency per se but rather reflects the allocation of attention towards the part of an object that signifies the object’s identity/function (e.g., an axe is perceived as such because of its blade, not its handle), and the potentiation of an action related to the long-term use of the object (Kourtis and Vingerhoets, 2015) and/or possibly the direction towards such action is performed (Pellicano et al., 2010).

This finding is an apparent contradiction with previous studies, which showed that the display of a graspable object may potentiate an action compatible with the immediate grasping of the object (Ellis and Tucker, 2000; Tucker and Ellis, 1998; Vainio et al., 2007). However, this effect is prominent when the observers attend to a feature of an object, which was, even indirectly, related to the handling of the object (Pellicano et al., 2010; Symes et al., 2007; Tipper et al., 2006), but it does not manifest when attention is directed away from the object (Vainio et al., 2007) or toward a feature that has nothing to do with the handling of the object, such as its color (Pellicano et al., 2010; Tipper et al., 2006). The participants in our study were instructed to simply attend an object as a whole, without paying particular attention to any of the objects’ grasping-related features, which would explain our behavioral results. It should be noted that the absence of behavioral evidence does not necessarily mean that the location of the handle of the object did not potentiate a grasping action, but it is possible that, similar to our previous study (Kourtis and Vingerhoets, 2015), this effect was rather weak and transient, and it was overshadowed by the stronger functional affordance effect.

The second behavioral effect was related to the size of the displayed object. The participants were faster responding with a type of grip that was the correct one for grasping the displayed object (e.g., with a precision grip when viewing a small object). This finding is in agreement with previous studies that have demonstrated that the size of an object potentiates a type of grasping action that is congruent to the object’s size (Ellis & Tucker, 2000; Grèzes, et al., 2003; Derbyshire et al., 2006). Notably, this effect was present only for responses made with the small switch but not with the large one. This could have been partly due to mechanical reasons, because the pressing of the large switch was a rather cumbersome and long-lasting action, but it is more likely that it predominantly reflects the fact that the participants responded faster to an arrow that was overlaid on a small object. The opposite effect was reported by Grèzes et al. (2003); those responses were faster when the stimulus was a large object. However, the participants in that study responded to the object itself (i.e., judging whether the object was manufactured or natural), whereas in the present study the participants responded to the direction of an arrow that was displayed on the object. A likely explanation is that large objects may have acted as sources of distraction and that might have delayed the detection of the arrowhead. Nevertheless, faster responses for small objects correspond to faster size-congruent responses with the small switch and faster size-incongruent responses with the large switch. As a consequence, the size-related congruency effect was enhanced for responses made with the small switch, but it was completely disguised (although not reversed) for responses made with the large switch.

A couple of limitations in the selection of the stimuli need to be acknowledged. First, the size of each object corresponded to the size of its grasping part (i.e., a large object had a large grasping part and a small object had a small grasping part). It is possible that the size congruency effect might not reflect the correspondence of the type of a grip to the size of the whole object but only to the size of its grasping part. Second, another factor to consider is the weight of an object, which in everyday life often is associated with its size (i.e., large objects are usually heavier that small objects). Similar to other object properties, the weight of an object is represented in the brain, and such representations involve the activation of motor areas (Chouinard et al., 2009; Jenmalm et al., 2006). However, the selection of our stimuli does not allow us to make dissociations between the object’s size and either the size of its graspable part or the object’s weight. This could be the topic of further investigations that may include objects, the size of which would not correspond to the size of their grasping part (e.g., small objects held with a power grip) or to the objects’ weight (e.g., small objects made of dense material).

Another parameter of our design that may have affected the magnitude of the two affordance effects is the duration of the interval that an object was displayed for. There is behavioral and neurophysiological evidence that affordance effects develop over time and reach their maximum when an object is displayed for 1,000 or 1,200 ms (Phillips and Ward, 2002; Vingerhoets et al., 2009b). However, recent work challenges these findings and suggests that affordance effects are short-lived and peak as early as 400 m after object display onset (Makris et al., 2011, 2013). For this reason, we performed an exploratory analysis on the RTs by dividing our data on the basis of the duration of the display interval (i.e., 500, 750, 1,000, 1,250, and 1,500 ms). Although the small number of responses per display interval prevents us from drawing safe conclusions, we found that each affordance effect was present in short and long intervals. We believe that the reason between the discrepant results with regards to Makris and colleagues’ studies may lie in the different task requirements. The participants in the present study prepared to respond to an arrow that appeared directly on the object, thus attending (features of) the object, whereas in the Makris et al. (2011) study, in which the effect of the duration of the display interval was explicitly investigated, the requirement for the participants was to respond to a change of the screen’s background color, which might have directed the participants’ attention away from the object. Certainly, this is a question that requires systematic investigation and cannot be resolved by the present findings.

### Neuroimaging results: distinct representations of function- and size-related affordances

All four main contrasts (SC > SInC, SInc > SC, FC > FInC and FInC > FC) resulted in activation of the extrastriate cortex (BA18 and BA19). This was an anticipated finding, because this area is generally associated with object identification and processing of object shape and location (Grill-Spector and Malach, 2004; Kourtzi and Kanwisher, 2001; Schwarzlose et al., 2008; Vingerhoets and Clauwaert, 2015; Whatmough et al., 2002). On the other hand, there was a clear distinction in the representation of function- and size-related affordances, the former being represented in the posterior parietal cortex (PPC) and the latter in prefrontal, premotor and primary sensorimotor areas.

The contrast of function congruent over function incongruent responses, which corresponds to the behavioral “function congruency effect,” revealed two activation clusters in the left PPC: one in the superior parietal lobule (SPL, BA 7), and a much larger activation cluster in the inferior parietal lobule (IPL, BA 40). Research on nonhuman primates has provided strong evidence that areas within the PPC; namely, the medial intraparietal (MIP) area and the anterior intraparietal (AIP) area have a central role in the organization of reaching and grasping movements, respectively (Andersen and Buneo, 2002; Grefkes and Fink, 2005). Although anatomical and physiological differences between nonhuman and human primates demand cautious interpretation of analogous findings across species, converging evidence suggests that the human homologues of these areas lie mainly along the intraparietal sulcus (IPS) but also within the SPL (for a review, Vingerhoets, 2014). The SPL is typically associated with action selection (Grafton et al., 1998) and online movement programming (Striemer et al., 2011), and it is believed to be part of the dorso-dorsal pathway (Binkofski and Buxbaum, 2013). The IPL sits on the opposite bank of the IPS, and it is a critical area in motor attention (Rushworth et al., 2003), representation of skilled movements (Haaland et al., 2000), planning and execution of object/tool related actions (Johnson-Frey et al., 2005; Randerath et al., 2010), and also in the representation of object affordances (Grèzes and Decety, 2002). The IPL is associated with the representation of grasping (Binkofski et al., 1999; Grafton et al., 1996), but it is probably not involved with the online-control of movement. Instead, it is believed to be part of the ventro-dorsal pathway, which is involved in the representation of long-term functional affordances, possibly by storing conceptual knowledge that may subserve the purposeful manipulation of object (Buxbaum et al., 2006; Valyear et al., 2007; Vingerhoets et al., 2009a). These functional roles of the SPL and of the IPL are consistent with the concept of “stable” and “variable” affordances. The IPL is considered to belong to a network that represents actions related to time-invariant features of an objects, whereas the SPL is mostly associated to online interactions with the object (Sakreida et al., 2016).

The opposite contrast (FInC > FC) showed two small clusters of activation, one in the left posterior cingulate gyrus (pCG, BA 23) and another one in the left (anterior) cerebellum. The pCG has been shown to be involved in object identification (Ellis et al., 2006) and also in visuospatial processing and orientation (Aguirre et al., 1996; Vogt et al., 2006). The cerebellum is a structure with an integral role in sensorimotor control and rapid movement prediction (Blakemore and Sirigu, 2003; Stoodley and Schmahmann, 2009). Moreover, research on the somatotopic organization of the cerebellum has revealed that the activation of the IV and V lobes (as in our study) is associated with the representation of hand-performed tasks (Nitschke et al., 1996). Thus, the increased pCG and cerebellar activation may reflect the representation of the identity as well as of the location of the object with respect to the participant’s hand and the automatic activation of a rapid movement towards the graspable part of the object.

The contrast of size congruent over size incongruent responses, which corresponds to the behavioral “size congruency” effect, did not involve the PPC, but instead it revealed activations in the prefrontal cortex (right BA9 and medial BA8). The prefrontal cortex is associated with decision making and also with object-based attentional selection activation (Cho et al., 2012; Coull et al., 1998; Hou and Liu, 2012); therefore, its involvement may reflect the participants’ decision to act upon the object with a size congruent grip. This interpretation is supported by the increased activations in size-congruent trials of the SMA proper and of primary sensorimotor areas, generally associated with late-stage movement selection, planning, and execution (Lee et al., 1999; Nachev, et al., 2007) and, related to our experimental design, with hand reaching and object grasping and manipulation (Hoshi and Tanji, 2004; Kuhtz-Buschbeck et al., 2001).

The opposite contrast (SInC > SC) showed one cluster of activation in the left middle frontal gyrus and more specifically in the left dorsal premotor cortex (PMd) (Mayka et al., 2006). Grèzes et al. (2003) have reported that the left PMd is involved in the implicit representation of object size affordances. Furthermore, PMd activity has been related to the accuracy of a (grasping) movement and also on the size of object that a person intents to grasp (Castiello and Begliomini, 2008; Grafton, 2010).

Taken together, our fMRI analysis shows that functional and structural action affordances that are related to different features of the same object are represented concurrently and to a large extent in distinct brain areas. Action affordances that are related to the identity/function of an object seem to involve the PPC and especially the IPL (see FC > FInC contrast), which is a structure associated with the long-term storage of skillful actions (Binkofski and Buxbaum, 2013; Sakreida et al., 2016). In addition, the involvement of the pCG and the cerebellum (see FInC > FC contrast) suggests that the location of the object’s handle may have activated a spatially compatible reaching action. The argument that the functional and the graspable end of an object could activate two competing response codes, although cannot be unequivocally ascertained by the present data, agrees with the findings of our recent EEG study (Kourtis and Vingerhoets, 2015), which also showed a precedence of the function-related effect.

Whereas representations related to the skillful use of the object and to a reaching movement toward the object seem to be mostly formed in the PPC, the actual shaping of the hand that matches the size of the object involves prefrontal, premotor and primary sensorimotor areas. Interestingly with regards to activation of premotor areas, the SMA was visible in the SC>SInC contract, whereas the PMd in the opposite one. The activation of the SMA is consistent with the notion of a “variable” affordances network (Sakreida et al., 2016). It is likely that reflects the updating of hand shape on a trial-to-trial basis, by initiating the motor program that fitted the size of the displayed object and possibly inhibiting the motor program that was not required on a given trial (Nachev et al., 2007). With regards to the Pmd activation in the SInC>SC contrast, work on primates have demonstrated that two actions can be concurrently represented in different neuronal population within the PMd (Cisek and Kalaska, 2002). In the present study, it is likely that both grasping actions were initially represented in the PMd and that the display of an object may have activated neuronal populations that coded a grasping action that was congruent to the object’s size. However, the direction of the arrow in size incongruent trials prompted the immediate activation of the complementary grasping action. Given the well-established role of the PMd in online movement control and adaptation (Lee and van Donkelaar, 2006; Ward et al., 2010), we propose that the increased PMd activation in size-incongruent trials may reflect the switch from the initially activated motor program to the one that was required for the performance of the task in a given trial.

Finally, it is worth pointing out that the majority of the activation clusters were located in the left hemisphere, which is in accordance with the view that planning and execution of object-directed actions are supported by a left-lateralized brain network, irrespective of the hand that performs the action (Johnson-Frey, 2004; Króliczak and Frey, 2009; Vingerhoets et al., 2012).

In summary, our study demonstrates that individual features of a graspable object may induce different type of action affordances even when the perceiver has no intention to act upon the object. These action affordances could be related to the long-term skillful use and also to the immediate grasping of the object. Our fMRI results show that different action affordances can be represented concurrently and to a large extent in distinct locations within the brain.

## Footnotes

1 We believe that the reason behind this interaction lies on the fact that responses were faster (~6 ms) when the arrow was overlaid on a small object compared with a large one (see *Discussion*).
